# Mechanism of intermediate filament recognition by plakin repeat domains revealed by envoplakin targeting of vimentin

**DOI:** 10.1038/ncomms10827

**Published:** 2016-03-03

**Authors:** Claudia Fogl, Fiyaz Mohammed, Caezar Al-Jassar, Mark Jeeves, Timothy J. Knowles, Penelope Rodriguez-Zamora, Scott A. White, Elena Odintsova, Michael Overduin, Martyn Chidgey

**Affiliations:** 1 School of Cancer Sciences, University of Birmingham, Birmingham B15 2TT, UK; 2 Institute of Immunology and Immunotherapy, University of Birmingham, Birmingham B15 2TT, UK; 3 Nanoscale Physics Research Laboratory, School of Physics and Astronomy, University of Birmingham, Birmingham B15 2TT, UK; 4 School of Biosciences, University of Birmingham, Birmingham B15 2TT, UK; 5 Institute of Cancer and Genomic Sciences, University of Birmingham, Birmingham B15 2TT, UK

## Abstract

Plakin proteins form critical connections between cell junctions and the cytoskeleton; their disruption within epithelial and cardiac muscle cells cause skin-blistering diseases and cardiomyopathies. Envoplakin has a single plakin repeat domain (PRD) which recognizes intermediate filaments through an unresolved mechanism. Herein we report the crystal structure of envoplakin's complete PRD fold, revealing binding determinants within its electropositive binding groove. Four of its five internal repeats recognize negatively charged patches within vimentin via five basic determinants that are identified by nuclear magnetic resonance spectroscopy. Mutations of the Lys1901 or Arg1914 binding determinants delocalize heterodimeric envoplakin from intracellular vimentin and keratin filaments in cultured cells. Recognition of vimentin is abolished when its residues Asp112 or Asp119 are mutated. The latter slot intermediate filament rods into basic PRD domain grooves through electrosteric complementarity in a widely applicable mechanism. Together this reveals how plakin family members form dynamic linkages with cytoskeletal frameworks.

## Abstract (web summary)

Plakin proteins link cell junctions to cytoskeletal frameworks, and their disruption within epithelial and cardiac muscle cells cause skin blistering diseases and cardiomyopathies. Here the authors use structural biology approaches to reveal the mechanism that allows plakins to recognize intermediate filaments within the cytoskeleton.

## Graphical abstract

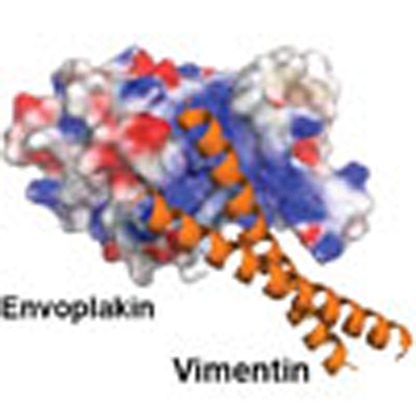

The cornified envelope is an essential component of the epidermal permeability barrier of vertebrate organisms. Once assembled under the plasma membrane of keratinocytes in the outer epidermis it prevents fluid loss from the body. The plakin protein envoplakin is upregulated during keratinocyte terminal differentiation, and interacts with the closely related protein periplakin to initiate cornified envelope formation^1^. Both proteins associate with desmosomes, the interdesmosomal plasma membrane and intermediate filament cytoskeleton. Together they represent a paradigm for how plakin proteins assemble onto intermediate filaments to create strong yet dynamic cytoskeletal anchoring for cell adhesion complexes. Other plakin proteins include the desmosomal protein desmoplakin, the hemidesmosomal proteins plectin and bullous pemphigoid antigen 1 (BPAG1, also known as dystonin), microtubule-actin cross-linking factor 1 (MACF1) and epiplakin^2^. Although their architectures have diverged, all plakin proteins except periplakin contain at least one plakin repeat domain (PRD), which constitutes a conserved node that links cell junctions directly to cytoskeletal frameworks, disruption of which has dire physiological consequences.

Envoplakin and periplakin are targeted by autoantibodies in the mucocutaneous skin-blistering disease paraneoplastic pemphigus, which accompanies malignant and benign neoplasia^2^. The majority of these patients express antibodies against envoplakin or periplakin C-terminal sites^3^. Such antibodies cause dissociation of cultured keratinocytes by an obscure mechanism that involves disruption of cell–cell adhesion^4^. Other plakin proteins also play vital roles in maintaining epidermal integrity. Plectin and BPAG1 are essential components of hemidesmosomes, and mutations in either protein cause the skin-blistering disease epidermolysis bullosa simplex^2^. Mutations in desmoplakin compromise the integrity of the skin and heart, and can lead to arrhythmogenic right ventricular cardiomyopathy, a common cause of cardiac arrest and sudden death in competitive athletes^2^,^5^. No mechanism-based therapies for these disorders are available, and only a few of the molecular impacts of mutations and antibodies have been explored^6^. Thus plakin proteins represent crucial elements for epidermal and cardiac muscle integrity, and understanding how they work could inform multiple clinical diagnostic and intervention strategies.

Plakin proteins engage intermediate filaments through PRD domains. Although their respective mechanisms are undefined, three subclasses of PRD modules have been identified as types A, B and C^7^. Desmoplakin contains one of each of the three PRD module types, which could each offer different functional specializations^7^. Of all the plakin proteins, envoplakin is the only member to contain a single PRD module, which is connected to a central coiled coil region through a linker domain (Fig. 1). The coil dimerizes with periplakin to form a stiff rod that connects to the hinged plakin domain, and thus to the membrane^8^.

As envoplakin is unique in possessing only one PRD module, we supposed that its singular PRD could incorporate all of the critical features needed to form strong links to intermediate filaments. Moreover, as its partner periplakin lacks any PRD module, envoplakin's PRD could tether the entire heterodimeric complex to the cytoskeleton^9^. While apo-structures of the PRD-B and -C domains of desmoplakin have been solved^10^, the structural basis of complex formation and specific cytoskeletal tethering has yet to be experimentally determined. To establish how PRDs recognize intermediate filaments we solved the envoplakin PRD structure by X-ray crystallography and small angle X-ray scattering (SAXS). The structure reveals a basic groove that can accommodate acidic patches on the cylindrical surface of monomeric or multimeric intermediate filament proteins. Complementary interacting residues within the groove and on vimentin's rod are revealed by point mutations and nuclear magnetic resonance (NMR) spectroscopy. Residues within the groove are shown to be important for targeting envoplakin to vimentin and keratin networks in cells. The pattern of conservation of key determinants across the plakin family infers a universal mechanism for plakin protein binding to the intermediate filament cytoskeleton in epithelia, cardiac and skeletal muscle.

## Results

### Structure of the envoplakin PRD

**Figure 2:**
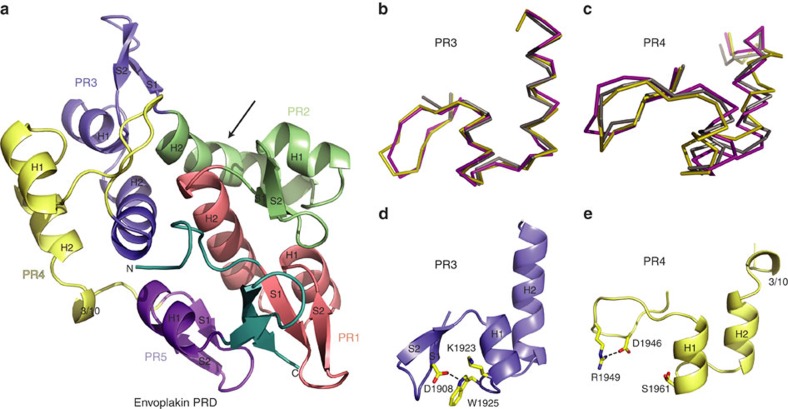
Distinguishing features of the envoplakin PRD structure. (**a**) Ribbon representation of the envoplakin PRD. Plakin repeats 1–5 are coloured red, green, blue, yellow and purple, respectively, with their PR motifs and secondary structural elements labelled. The N and C termini are labelled and shown in teal. The arrow indicates the kink of H2 in PR2. (**b**) Overlay of PR3 from envoplakin (magenta) and desmoplakin PRDs B (olive) and C (grey). (**c**) Overlay of PR4 from envoplakin and desmoplakin PRDs B and C. (**d**) Stabilization of the envoplakin PR3 by hydrogen bonding (black-dashed lines). (**e**) Stabilization of PR4 by hydrogen bonding.

To elucidate the mechanism of intermediate filament recognition, we determined the complete structure of envoplakin's PRD module (Fig. 2a; Table 1). The construct boundaries were selected based on sequence alignment (Supplementary Fig. 1a), and the resulting structure was solved to 1.6 Å resolution by single-wavelength anomalous dispersion. The asymmetric unit was comprised of two PRD molecules designated ‘a' and ‘b'. Models comprising residues Asp1822 to Ala2014 were built and refined to *R* and *R*_free_ factors of 16.8% and 20.5%, respectively. The electron density was unambiguous for the entire polypeptide chain, resolving previously undefined features. The protein was exclusively monomeric as judged by sedimentation equilibrium experiments at 21, 44 and 65 μM, respectively (Supplementary Fig. 2a), and sedimentation velocity experiments at 46 μM. SAXS studies also indicated a single, monomeric state with an oblong shape (Supplementary Fig. 2b,c). The two envoplakin PRD molecules in the asymmetric unit are similar, superimposing with a root mean square (r.m.s.) deviation of 1.1 Å for 183 aligned Cα atoms. The deviations mainly originate from lattice contacts. As they are similar both molecules a and b fit equally well into the SAXS envelope (Supplementary Fig. 2c). Therefore, molecule a (hereafter referred to as the envoplakin PRD) was used for subsequent analyses.

### Distinctive structural features of envoplakin's PRD

The envoplakin PRD structure has two lobes and contains 4.5 copies of the canonical 38 amino-acid plakin repeat (PR) motif followed by a C-terminal hairpin turn (Fig. 2a). Each motif is comprised of an 11 residue β-hairpin followed by an antiparallel pair of α helices. The overall fold of the envoplakin PRD is most similar to the structures of the isolated desmoplakin PRD-B (PDB: 1LM7) and PRD-C (PDB: ILM5) constructs that were resolved previously at 3.0 and 1.8 Å, respectively^10^. Most similar was desmoplakin PRD-C, with which envoplakin exhibits a backbone r.m.s. deviation of 1.6 Å for 186 aligned Cα atoms. All the PRD structures are congruent, with the irregular PR2 and PR4 helical features being most divergent structurally. All cases present two lobes comprised of the closely packed PR1-PR2 and PR3-PR4 motif pairings, respectively, suggesting that the PRD domain may have originally emerged from duplication of tandem PR motifs. The core interactions and loop features revealed in envoplakin's PRD structure appear to be conserved throughout envoplakin's evolution inferring functional importance. Structural heterogeneity is found in between PR3 and PR4, and involves the pinning of their β-hairpins into place (Fig. 2b–e). In desmoplakin PRD-B and PRD-C, these elements are predominantly stabilized by ion-pair interactions formed by an Asp residue projecting from the S1 strand (PR position 4) and a positively charged residue protruding from the H1 helix (PR position 19). Envoplakin's β-hairpins differ, despite the fact that their PR elements (except for PR4) possess the relevant charges at these positions. This is exemplified in PR3 where Asp1908 and Lys1923 are orientated away from each other, their missing ion-pair interaction being offset by a hydrogen bond between Asp1908 and Trp1925 (Fig. 2d). Indeed, this arrangement and the presence of nearby lattice contacts may contribute to the resolution of envoplakin's PR3 β-hairpin (Supplementary Fig. 3a–c). In contrast, the corresponding β-hairpin of desmoplakin PRD-C (Glu2699 to Lys2705 in chain A, and Lys2702 to Lys2704 in chain B) appears disordered presumably due to a combination of a loss of ionic interaction resulting from a non-conservative substitution at PR positions 4 (Gly2700) and 19 (Glu2715) and an absence of lattice contacts here^10^. The lack of defined β-hairpin structure in PR3 of desmoplakin PRD-C has consequences for the anti-parallel arrangement of the H1 and H2 helices, which are closely paired rather than splayed as in envoplakin. A novel conformation is found in envoplakin's PR4 motif, where a long loop is found instead of the canonical β-hairpin. The lack of regular secondary structure here may be influenced by Lys2753 in desmoplakin PRD-C being replaced by Ser1961 in the corresponding position of envoplakin, which hence cannot form the salt-bridge interaction that fixes the canonical β-hairpin in place. However, a compensatory interaction between Asp1946 and Arg1949 in PR4 may contribute to the irregular structure of this envoplakin region (Fig. 2e). Finally, the envoplakin PRD exhibits a 3_10_ helix in PR4 from which Glu1975 protrudes to contact the PR3 and PR5 helices, whereas the corresponding desmoplakin region adopts no regular secondary structure. That this element is highly conserved in envoplakin orthologs but diverges widely between the PRD superfamily members (Supplementary Fig. 1a,b) suggests a key role here in relative PR positioning within the domain.

A striking feature observed in envoplakin is the non-canonical helical structure and distinctive kink of H2 in PR2 (Fig. 2a; Supplementary Fig. 3d). Its significance can now be inferred by contrasting this feature with the corresponding desmoplakin elements, as it consistently acts as a junction between segments that pack within and between PR motifs, respectively. The deviation from regular α-helical character here appears to be generated by two distinct mechanisms. In desmoplakin PRD-C, Pro2690 is responsible for distorting the regular geometry midway through the helix. Notably, such proline-induced kinks are hallmarks of transmembrane helices that require flexibility for movement between the signalling states^11^. In contrast, the corresponding positions of the envoplakin PRD and desmoplakin PRD-B modules are occupied by Asn1898 and Ser2331, respectively. This reveals that a proline residue is not strictly required here for kinking, as a shift in hydrogen-bonding pattern between the donors and acceptors is consistently found in the H2 bend. Moreover, in all PRDs this helical distortion is maintained by favourable side-chain interactions mediated by residues comparable to envoplakin Gln1900, Ala1902, Phe1903, Val1879, Gln1938 and Gly1866. That this kink architecture has been so well conserved between PRD family members despite the sequence variability here suggests that the PR2 helical bend makes a critical contribution, as is supported by neighbouring functional residues (see below).

Whether flexibility could contribute to the differences between envoplakin and desmoplakin was assessed by examination of disorder. The envoplakin PRD domain was uniformly ^13^C and ^15^N labelled, its backbone resonances assigned and the order parameters (*S*^2^) estimated using Talos+ (ref. 12). This revealed a uniformly ordered domain with elevated disorder in the N-terminal, PR2, PR3 and PR5 β-hairpin loop sequences (Supplementary Fig. 4a,b). Further disorder was apparent in the PR2 helices, H1 of PR4, and the H2 and 3_10_ helix of PR4. The flexibility of the N-terminal end, and the loops of PR3 and PR5, is further supported by comparatively large r.m.s. deviations between the two envoplakin molecules which comprise the asymmetric unit of the crystal structure, and extend to the desmoplakin PR2 and PR3 hairpin loops, and position of the 3_10_ helix based on consistently high B-factors (Supplementary Fig. 4c,d). Together this maps out cross-validated dynamic elements that encircle a major groove, as would be consistent with a large binding site. Underneath the relatively dynamic surface formed by PR2, PR3 and PR4 elements is the PRD core which is stabilized by a single continuous network of buried hydrophobic contacts that integrates all PR motifs. The alignment reveals that envoplakin's core residues are highly conserved across the PRD family, with divergences in structural and dynamic elements suggesting specialized functional features (Supplementary Fig. 1).

### Vimentin recognition determinants within the PRD

**Figure 3:**
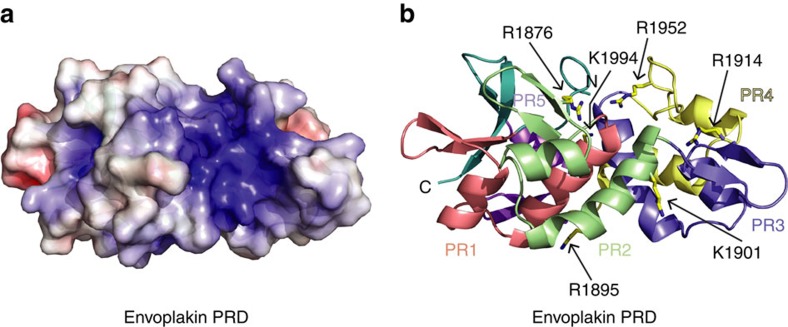
The envoplakin PRD contains a conserved basic groove. (**a**) Electrostatic surface potential of envoplakin PRD calculated with DelPhi. The potential scale ranges from −7 (red) to +7 (blue) in units of kT/e. (**b**) Ribbon representation of the envoplakin PRD highlighting the position of positively charged residues mutated in this study. The orientation is the same as in **a**, and the colours as in Fig. 2.

The electrostatic potential of the envoplakin PRD surface reveals a polarized protein with a highly positively charged groove (Fig. 3a). One basic flank is formed by H2 of PR1 and H2 of PR2, another by S1, S2 and H2 of PR3, while the elongated loop of PR4 forms a cap (Fig. 3a). This surface area contains a deep groove which is ∼23 Å long and 10 Å wide (Fig. 3a). The charge within its concavity is pronounced. Basic residues protruding from PR2 include Arg1876 in S2, and Arg1895 and Lys1901, which flank the kink in H2. Additional charges are contributed by Arg1914 in PR3:S2, Arg1952 in the extended PR4 loop, and Lys1994 from PR5:H1. The groove is delineated by Arg1952 and Lys1994 at one end, and Lys1901 and Arg1914 at the other extremity, while Arg1876 and Arg1895 lie centrally (Fig. 3b). Together this defines a major groove that could conceivably accommodate a large molecular cylinder. Its basic residues are conserved in desmoplakin PRD-C but less so in PRD-B^10^, and while its dimensions more closely resemble desmoplakin PRD-C, envoplakin's groove is slightly narrower albeit similar in length. Interestingly, the envoplakin PRD's lattice contacts involving PR3 overlap the large groove and could conceivably stabilize a conformation relevant to ligand binding. As no specific binding determinants have yet been established and co-crystals proved elusive, we sought to characterize the interactions by NMR.

Titrations were first carried out with full-length human vimentin protein (vimentin^FL^) in the absence of salt because vimentin forms filaments of heterogeneous lengths that are not suitable for characterization by NMR in the presence of salt^13^. In the absence of salt, vimentin^FL^ forms functional tetramers^14^. The optimized solution conditions used here allowed the progressive changes in resolved amide signals to be monitored as functionally relevant vimentin^FL^ tetramers were added stepwise into a ^15^N-labelled envoplakin PRD sample to respective molar ratios of 0.1:1, 0.5:1, 1:1 and 2:1. The interaction with the filaments exhibited slow exchange on the NMR timescale as evidenced by progressive line broadening. The effect on line shape was dramatic, with only 18% of the PRD amide peaks retaining at least 20% of their starting peak intensities at a half equimolar ligand concentration, with the remainder being around the detection limit (Supplementary Fig. 5a,b). Together this suggests that monomeric PRD modules directly assemble on vimentin^FL^ to form large, stable and slowly tumbling complexes, and may collectively decorate multiple sites on the filament.

To examine the roles of basic determinants in binding vimentin^FL^, several envoplakin PRD mutants were designed. These included alanine and charge reversal substitutions of the six arginine and lysine residues in the groove as well as control mutations of distal conserved basic residues. The similarity of the ^1^H,^15^N resolved spectra of the mutant and wild-type forms indicated that the proteins were folded (Supplementary Fig. 5a,c). Only the R1876E and R1952E variants yielded poor peak dispersion in heteronuclear single quantum coherence spectra, hence the functional contributions of these positions were inferred from the corresponding alanine substitutions, which did not compromise folding.

**Figure 4:**
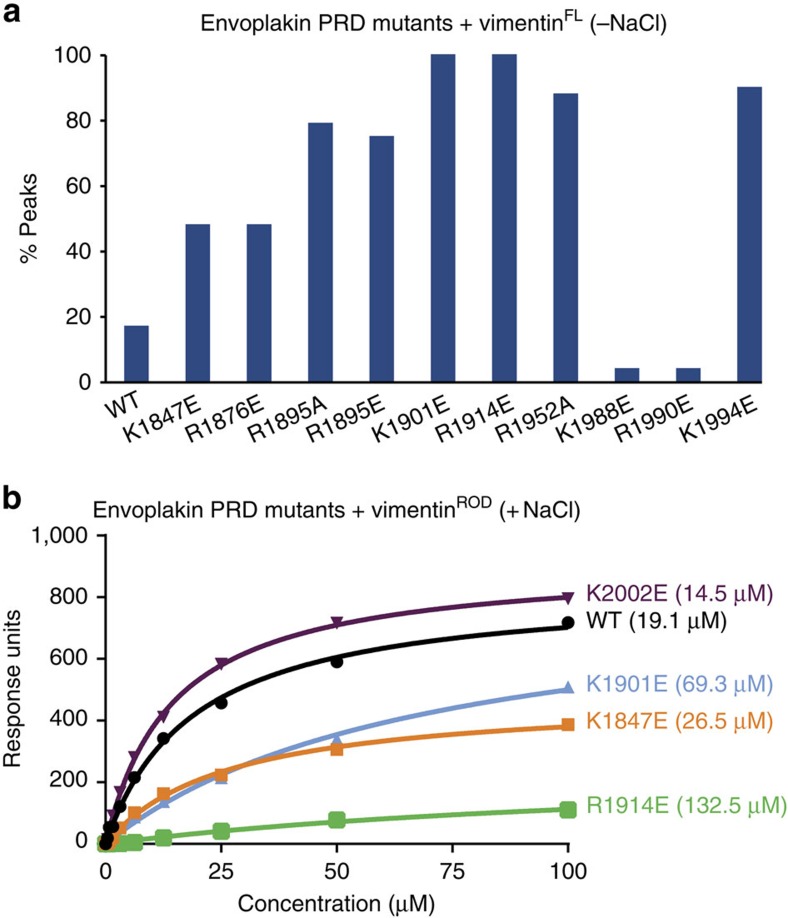
Mutating residues in envoplakin's basic groove abolishes vimentin binding. (**a**) Histogram showing the percentage of ^1^H,^15^N amide peaks retaining more than 20% of their peak intensity on addition of 50 μM full-length vimentin to wild-type (WT) and mutant envoplakin PRD proteins (100 μM) in the absence of salt. (**b**) SPR analysis of wild-type and mutant envoplakin PRD binding to vimentin^ROD^ in the presence of 150 mM NaCl. The figures in parentheses are *K*_D_ values. The data shown is representative of a number of experiments. *K*_D_ values were estimated to be: WT=19.1±1.3 μM, K1847E=26.5±2.0 μM, K1901E=69.3±10.6 μM, R1914E=132.5±34.7 μM and K2002E=14.5±0.4 μM.

Binding to vimentin^FL^ was compromised by the R1895E, K1901E, R1914E, R1952A and K1994E substitutions (Fig. 4a; Supplementary Fig. 5c,d). Control mutations K1988E and R1990E had no such deleterious effect on vimentin^FL^ interactions, while another control mutation, K1847E, decreased envoplakin PRD binding to vimentin^FL^ to a small extent (Fig. 4a). Overall, the data are consistent with vimentin^FL^ binding via the positively charged groove, and indicates the obligate involvement of each of these residues in tight binding of intermediate filaments. On the basis of the dramatic reductions in peak intensity, the R1895E, K1901E, R1914E, R1952A and K1994E positions play critical roles, consistent with the high conservation of their positive charges (Supplementary Fig. 1). The position of the critical Lys1901 residue in the highly conserved helical PR2 kink suggests a pivotal role in binding. The Arg1914 residue is found in a part of PR3 that displays dramatically different conformers in the different PRD structures and therefore may help in the induced fit of ligands. This groove region generally exhibits elevated disorder (Supplementary Fig. 4) suggesting a flexible character that could aid in binding. Together this defines the PRD function as providing specific determinants to form the bridges to intermediate filament sites for the cell adhesion machinery.

To exclude the possibility that non-specific electrostatic association was responsible for envoplakin PRD–vimentin interactions, a truncated vimentin construct was designed that, unlike vimentin^FL^, does not polymerize in the presence of physiological salt concentrations. This vimentin^ROD^ construct consists of residues 99–249 and encompasses coils 1A and 1B of the central rod domain. It is a stable monomer as evidenced by Analytical ultracentrifugation (AUC) in the presence of 150 mM NaCl (Supplementary Fig. 6a), and contains an estimated α-helical content of 68% based on analysis of its far-ultra violet circular dichroism spectrum using the CDSSTR algorithm and reference data set 7 on the DICHROWEB server^15^,^16^ (Supplementary Fig. 6b–d). Binding experiments with the vimentin^ROD^ construct were performed by surface plasmon resonance (SPR) with a BIAcore 3000 instrument in the presence of 150 mM NaCl (Fig. 4b). A binding constant of 19.1 μM was obtained for wild-type envoplakin PRD binding to the vimentin^ROD^ protein. Interactions of the K1901E and K1914E mutants were much weaker (69.3 and 132.5 μM, respectively), while the binding affinity of two control envoplakin PRD mutants K1847E and K2002E was comparable to wild-type (26.5 and 14.5 μM, respectively). Similar results were obtained with our NMR binding assay (Supplementary Fig. 5e,f). Overall, the SPR and NMR binding data validate the predominantly electrostatic binding mode of the envoplakin PRD for its vimentin ligand, whether presented as a stable tetramer in low salt or in a monomeric form in physiological salt.

### Determining envoplakin's intracellular distribution

**Figure 5:**
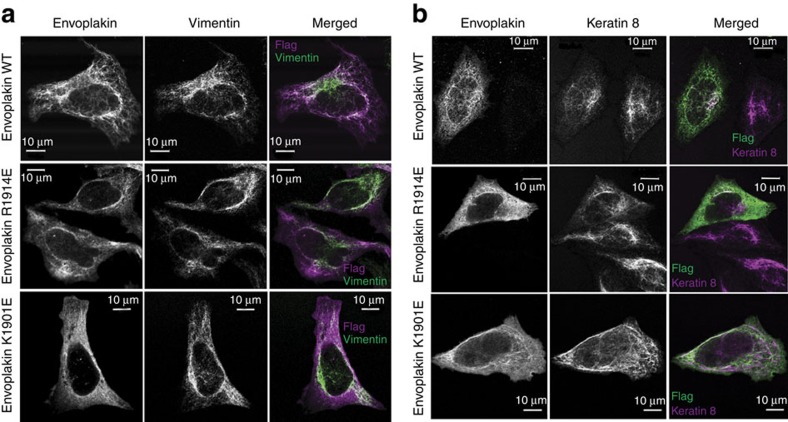
Intracellular distribution is compromised on mutating envoplakin's basic groove residues. Constructs encoding residues 1542–2014 of human envoplakin with a C-terminal FLAG tag and residues 1588–1756 of human periplakin with a C-terminal HA tag (Supplementary Fig. 6a) were co-transfected into HeLa cells. The cells were stained with anti-FLAG (against envoplakin) and either (**a**) anti-vimentin or (**b**) anti-keratin 8 antibodies, showing the loss of cytoskeletal localization caused by the R1914E and K1901E mutations.

To establish the biological role of the basic binding groove, we tested the effects of envoplakin mutations on co-localization with intermediate filaments in cultured HeLa cells. Two mutations, K1901E and R1914E, were selected as residues 1,901 and 1,914 are exposed but on opposite sides of the binding groove. Neither K1901 nor R1914 plays a significant structural role and both positions are highly conserved in envoplakins (Supplementary Fig. 1b). The K1847E and K2002E mutations were chosen as controls due to their exposed positions outside the binding groove. When expressed alone the envoplakin C-terminal construct mainly exhibits a punctate cytoplasmic distribution, as expected from previous studies^9^, whereas it shows extensive co-localization with vimentin when co-expressed with a matching periplakin construct with which it pairs (Fig. 5a; Supplementary Fig. 7a). In contrast, when envoplakin K1901E and R1914E mutants were co-expressed with periplakin they only partially co-localized with vimentin, the majority being cytosolic and diffuse (Fig. 5a). Mutants K1847E and K2002E showed identical patterns of staining to wild-type envoplakin (Supplementary Fig. 7b), confirming that the effect was specific for residues located in the groove. The intracellular distribution of periplakin was unaffected, displaying co-localization with vimentin when expressed with wild-type, K1901E or R1914E mutant envoplakins (Supplementary Fig. 7c). All four envoplakin mutants showed similar levels of expression to wild-type envoplakin, and all four mutants bound periplakin in pull-down assays (Supplementary Fig. 7d). The co-localization of wild-type envoplakin with keratin intermediate filaments was extensive, whereas that of the K1901E and R1914E mutant envoplakins was more diffuse (Fig. 5b). Altogether this indicates that the K1901E and R1914E mutations compromise envoplakin's ability to co-localize with intermediate filaments, establishing the PRD groove as being responsible for cytoskeletal contact by cytosolic plakin protein assemblies.

### Structural model of the envoplakin PRD–vimentin complex

Vimentin forms rod-shaped multimeric structures which offer multiple acidic patches (Supplementary Fig. 8) that could be recognized by the basic PRD groove. The assemblies were modelled using High Ambiguity Driven DOCKing (HADDOCK) for experimentally restrained protein–protein docking^17^. The envoplakin residues shown here to be involved in vimentin binding including Arg1895, Lys1901, Arg1914, Arg1952 and Lys1994 were used to restrain docking to conserved negatively charged surfaces within the vimentin rod domain.

**Figure 6:**
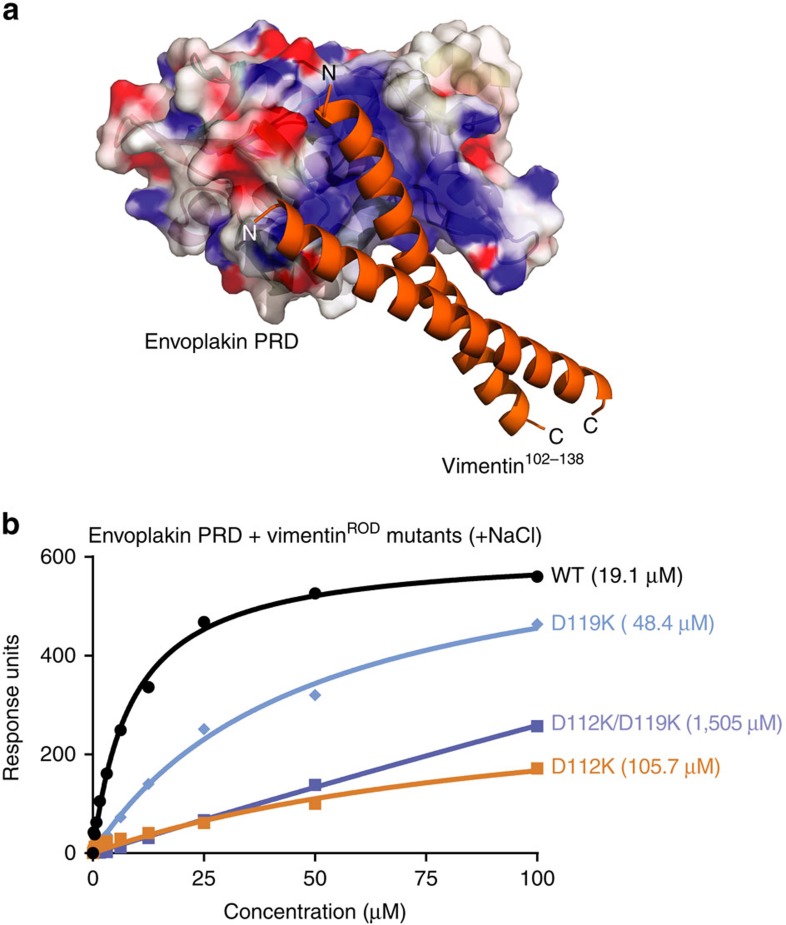
Modelling and binding studies of the envoplakin PRD–vimentin complex. (**a**) The envoplakin PRD structure (coloured as in Fig. 3) was docked with vimentin Asn102-Leu138 (orange) (PDB 3G1E) using HADDOCK. (**b**) SPR analysis of wild-type envoplakin PRD binding to wild-type and mutant vimentin^ROD^ in the presence of 150 mM NaCl. The figures in parentheses are *K*_D_ values in μM. The data shown is representative of a number of experiments. *K*_D_ values were estimated to be: WT=19.1±1.3 μM, D112K=105.7±45.6 μM, D119K=48.4±5.5 μM and D112K/D119K=1505±139 μM.

The resulting models converged, despite starting with different vimentin structures including both monomeric and dimeric states. Vimentin consistently slotted into the positively charged groove identified in the envoplakin PRD with minimal structural rearrangement. The major point of divergence was the angle of vimentin entry and egress from the groove (Fig. 6a), as expected given the multiple available vimentin binding surfaces that could be satisfactorily oriented within broad groove. While several of vimentin's acidic patches mediated favourable interactions, the model with the lowest energy consisted of the PRD contacting vimentin's helical Asn102-Leu138 sequence (Fig. 6a; Supplementary Table 1). The model suggests that direct interactions are mediated by residues Lys104, Asn111, Asp112, Asp119 and Lys120 in chain A and Glu106, Arg113 and Glu125 in chain B of the coiled coil dimer (PDB code 3G1E)^18^. Strikingly, in all models examined, Lys1901 and Arg1914, which line the bottom of the groove, appear to form contacts with conserved vimentin residues. In contrast, Arg1895, Arg1952 and Lys1994 played peripheral roles within the interface, consistent with the intermediate effects of their mutations as seen by NMR. Additional PRD groove residues which were proximal to vimentin include Glu1875, Lys1881, Arg1885 and Lys1948, which are only moderately conserved. As such, these positions could help generate specific complementarity of PRD variants for diverse intermediate filament ligands. The overall compatibility of the binding model infers a shared cytoskeletal recognition mechanism in which acidic filament ridges fit into basic PRD grooves offered by plakin protein assemblies.

To validate the proposed mode of vimentin recognition, mutant versions of the vimentin^ROD^ construct were designed. This longer construct was required as the envoplakin PRD showed no detectable binding to a peptide consisting of vimentin residues 102–138 (equivalent to PDB 3G1E) by SPR in the presence of salt, presumably because of its inability to form a stably folded helical coiled coil. The affinity of wild-type envoplakin PRD for wild-type envoplakin^ROD^ was 19.1 μM, while its affinity for vimentin^ROD^ point mutants D112K and D119K was compromised (105.7 and 48.4 μM, respectively) and for a double mutant D112K/D119K was effectively abolished (*K*_D_=1.5 mM) (Fig. 6b). Hence vimentin's D112 and D119 residues appear to be crucial for recognition by the PRD. Nonetheless, other secondary sites cannot necessarily be excluded and are in fact likely based on the occurrence of similar motifs in intermediate filament structures, and the need for adaptive plakin protein engagement with the cytoskeleton.

### Wider implications for the PRD superfamily

**Figure 7:**
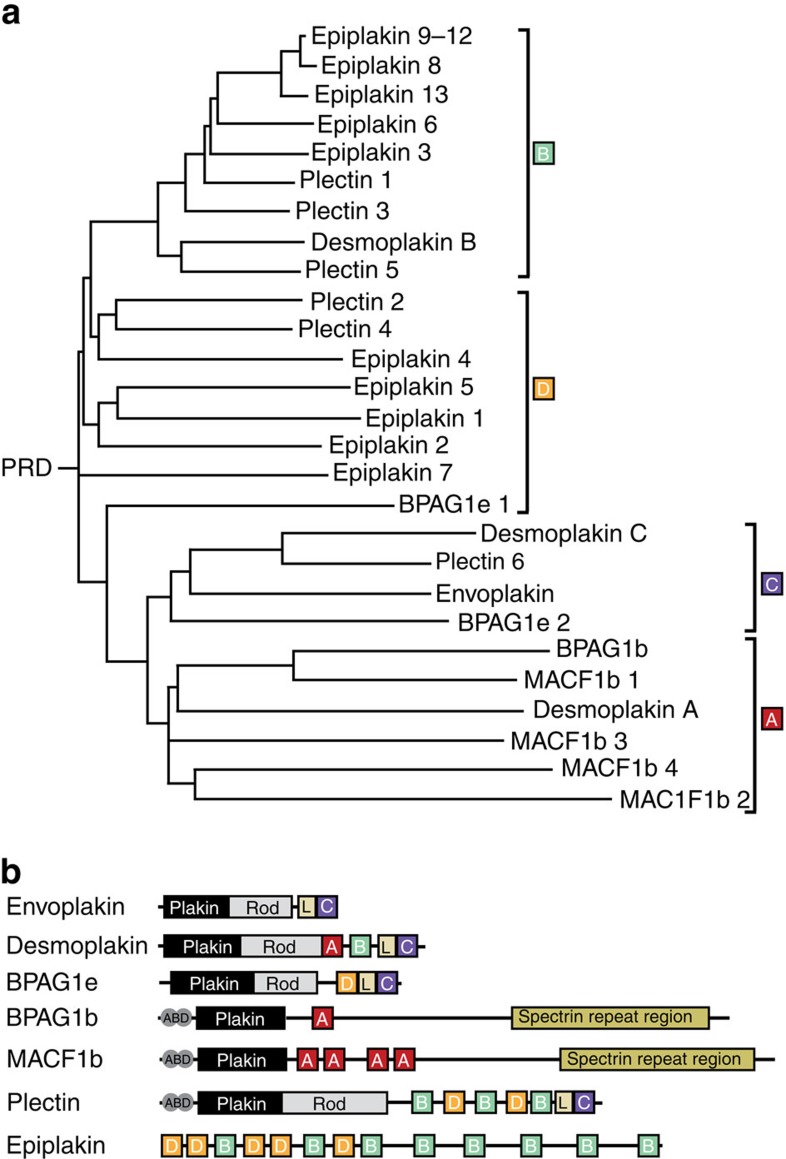
Structural and functional relatedness of human PRDs. (**a**) Phylogenetic tree of the PRD family including human envoplakin, desmoplakin, BPAG1e, BPAG1b, MACF1b, plectin and epiplakin, as generated by ClustalW. PRD modules are grouped into four subtypes, including a new PRD-D subtype. (**b**) Domain organization of the human plakin proteins with their PRD subtype and additional modules indicated. Note that BPAG1b and BPAG1e are encoded by the dystonin gene.

Comparison of the structural and functional motifs across all 29 human PRD sequences clarifies the functional architecture and reveals a novel type of subclass module in addition to the established PRD-A, -B and -C domains. A fourth major subtype is present only in BPAG1e, plectin and epiplakin. Their ‘PRD-D' modules are distinguished by divergent PR4–PR5 sequences that cap their grooves, and may have weaker intermediate filament affinities based on the lower basic character of their grooves (Fig. 7). In contrast essentially all PRD-C modules offer highly electropositive grooves that connect to the structurally independent linker domains. The PRD-A modules have few or no basic residues in their grooves, and may offer as of yet undiscovered functions. The PRD-B subgroup members contain moderately basic grooves, and lack the PR3 clamp feature found in PRD-C modules. Together this suggests that plakin proteins represent a structurally conserved family of cytolinkers that are adapted through functionally divergent PRD modules to assemble and dissemble the many filamentous networks responsible for maintaining the dynamic integrity of cellular architectures.

## Discussion

The cornified envelope presents vertebrate organisms with a remarkably durable yet adaptable layer of cross-linked protein that is formed in the outer epidermis and is vital for epidermal permeability barrier function. Key components include envoplakin which, together with the closely related protein periplakin, form a complex scaffold on which other proteins of the cornified envelope can be deposited^19^. Envoplakin is not just expressed in the epidermis, but is found in a variety of other epithelial tissues including those of mammary gland, bladder and stomach^20^, and this, together with its association with desmosomes at the cell periphery, strongly suggests that it has a role in intercellular adhesion with its PRD acting as a molecular linchpin connecting the membrane directly to the cytoskeleton.

The envoplakin PRD has five PR motifs, four of which project basic residues into a long groove, acting as digits that grasp negatively charged patches on vimentin filaments. PR3 projects an apparently dynamic β hairpin with a putative hinge formed by the absolutely conserved Gly1904 and Gly1918 residues. This conceivably allows clamping down on a bound filament by Arg1914 to lock a filament into position. Although structurally inconsequential, charge reversal mutations here disrupt interactions with vimentin in protein binding and cellular assays. The PRD groove presents a concave swathe of six basic residues that are required for binding tightly to vimentin, accommodating its cylindrical acidic surface. Mutation of single basic binding determinants reduces but does not completely abolish the ligand interaction, indicating that polarized curvature with a charged array is needed for filament attraction, although non-polar residues also contribute^10^. PRD domains from other plakin proteins contain different numbers of basic determinants, and given that intermediate filaments offer multiple potential binding sites with varying numbers of charged residues, a spectrum of weak to strong PRD binding to ligands may also be available. Further complexity is likely to be caused by variations in steric fit and non-polar contributions from the variety of available filaments and potential binding partners as well as 180° reverse orientations. Altogether this implies that PRD modules are presented with a wealth of opportunities for sliding and locking onto filaments, facilitating the dynamic assembly and disassembly of junctional complexes between epithelial cell architectures.

The determinants of plakin protein tethering to the cytoskeleton appear to be widely held. The major properties of envoplakin's long vimentin binding groove are conserved, with broadly similar diameters, lengths and charge distributions being presented by the envoplakin and desmoplakin PRD structures. The envoplakin PRD has flexible loop regions and structural elements surrounding the positively charged groove. This includes dynamic elements including the loop region of PR2, which is situated before R1876, H2 of PR2 which contains K1901, the loop region of PR3 which lies before K1914 and the H2-3_10_ helical region of PR4 which is situated behind the groove. These elements could guide ligand entry into the groove. The groove itself is capable of readily accommodating vimentin filaments, which have diameters of 6.7–23.6 Å and present multiple acidic patches on their cylindrical surfaces. Such polyacidic motifs are conserved in other intermediate filaments such as desmin, and keratins 1 and 10. This infers that plakin superfamily proteins could interact with various filament partners through a common binding mode in which a basic groove inducibly accommodates cylindrical ligands through complementary electrostatic interactions.

Although the envoplakin PRD is clearly capable of binding intermediate filaments, it appears that periplakin is largely responsible for targeting envoplakin–periplakin heterodimers to the cytoskeleton, consistent with earlier studies^9^. Thus, when transfected into cultured cells truncated envoplakin is distributed in a punctuate pattern throughout the cytoplasm but on co-expression with truncated periplakin it shows extensive cytoskeletal co-localization. Intriguingly, when truncated envoplakin containing R1914E and K1901E mutations is co-transfected with truncated periplakin, the mutant envoplakins show only partial co-localization with vimentin whereas periplakin retains its original staining pattern. Given that the truncated mutant envoplakin and truncated periplakin proteins interact, then how can the different distributions of the proteins be reconciled? We suggest that periplakin initially recruits K1901E and R1914E envoplakin to the cytoskeleton via heterodimerization, and that subsequently the mutant envoplakins are less likely to be retained in place, once a cytoskeletal localization has been achieved, because of their compromised ability to bind vimentin. Thus the envoplakin–periplakin–vimentin interaction may be dynamic, facilitating cytoskeletal reorganization and remodelling during processes such as wound healing and epidermal differentiation. The *in vivo* interactions are complex, with both envoplakin and periplakin containing linker domains that may also contribute to intermediate filament recognition subject to oligomerization, disulfide bond formation and phosphorylation^21^,^22^,^23^,^24^,^25^,^26^. Thus the PRD not only binds intermediate filament proteins but may also aid in assembly and disassembly of envoplakin–periplakin–intermediate filament complexes.

Our model provides a basis for understanding the mechanism by which plakin proteins containing multiple PRDs, such as desmoplakin and plectin, bind to intermediate filaments. That is, plakin proteins with multiple PRD modules bind to filaments such as vimentin which project multiple acidic patches along the length of their cylindrical surfaces. This would allow the proteins to engage dynamically at more than one location. Formation of larger plakin protein networks in cell–cell and cell–matrix junctions could allow further interactions with intermediate filament proteins, thus reinforcing the links. Such multivalent interactions could be required to confer the strong interactions required to maintain desmosomal and hemidesmosomal adhesion in epithelial tissues that are subject to mechanical stress.

Structure-based phylogenetic analysis suggests that functional differences exist between the different classes of PRD modules. The PRD-B and PRD-C subtypes generally possess highly basic binding grooves, and may bind more tightly to intermediate filament cytoskeletons than PRD-A and PRD-D subtypes, which tend to have lower net charges. Interestingly, similar PRD–linker–PRD modules are present in all three plakin proteins that are found in cell junctions that normally resist skin blistering (that is, desmoplakin, BPAG1e and plectin), and the tight interaction of plectin with intermediate filaments is mediated by this module^23^. Envoplakin contains a linker–PRD-C module and its binding to vimentin, as judged by SPR, is relatively weak. Thus it may be that in the case of envoplakin tight binding to the cytoskeleton (in the absence of a PRD–linker–PRD module) requires heterodimerization with its partner periplakin.

The PRD mechanism provides a rational basis for manipulating plakin proteins and their partners including vimentin, desmin and keratins. The role of envoplakin in cornified envelope formation and cell adhesion can now be more precisely probed and the specific effects of PRD mutations in disease can be explained. Sequencing of somatic cancers has identified 18 mutations in envoplakin's PRD including Y1831F, D1832Y, D1981N and R1999C^27^. While the biological consequences have yet to be established, these substitutions would have structurally deleterious effects. Other mutations, such as R1876C that has been found in an upper aerodigestive tract cancer^28^, would directly compromise intermediate filament binding. An array of mutations in desmoplakin PRD's have been mapped and may contribute to arrhythmogenic right ventricular cardiomyopathy (ARVC) pathogenesis^29^. It is now clear that those such as G2375R^30^ and R2639Q^31^,^32^ would introduce structural vulnerabilities into PRD folds. The effects of other mutations on complex formation can now be modelled, thus helping to translate genome-wide screening and patient data into specific molecular consequences.

## Methods

### Expression and purification of proteins

The wild-type human envoplakin PRD construct was designed based on the sequence conservation of its structural elements, and DNA encoding the domain (residues Asp1822–Ala2014) was synthesized by Shinegene Molecular Biotech (Shanghai, China) and cloned into the expression vector pProEX-HTC (Life Technologies). Envoplakin PRD mutants were produced by QuikChange Lightning site-directed mutagenesis (Agilent Technologies). DNA encoding the envoplakin PRD was transformed into BL21(DE3) cells. Bacterial cultures were incubated at 37 °C in either LB media or M9 minimal media supplemented with ^15^NH_4_Cl. The cells were grown until an OD_600_ of 0.6 was reached, the temperature was reduced to 18 °C and expression was induced with 1 mM isopropyl-β-D-thiogalactopyranoside (IPTG). Cells were collected by centrifugation after 18 h and resuspended in phosphate-buffered saline (PBS) with protease inhibitors (Roche). Cells were lysed using an EmulsiFlex-C3 (Avestin) and cell debris were removed by high speed (75,600*g*) centrifugation. The clarified supernatant was applied to a HisTrap column (GE Healthcare) that had been pre-equilibrated with 500 mM NaCl, 10 mM imidazole, 20 mM Hepes, pH 7.5. The column was washed with 500 mM NaCl, 30 mM imidazole, 20 mM Hepes, pH 7.5 and His-tagged protein eluted from the column using 500 mM NaCl, 250 mM imidazole, 20 mM Hepes, pH 7.5. Tobacco Etch Virus protease was added to the protein and the mixture dialysed in 500 mM NaCl, 20 mM Hepes, pH 7.5. The protein was applied to a pre-equilibrated HisTrap column to remove the cleaved His tag and further purified by size exclusion chromatography using an S75 column (GE Life Sciences). Fractions containing the PRD protein were concentrated using Amicon Ultra-15 Centrifugal Filter Concentrators (Millipore).

Full-length human vimentin (residues Met1-Glu466) was synthesized by ShineGene and cloned into vector pET21a+ (Novagen). The DNA was transformed into BL21 (DE3) cells and bacterial cultures grown as before. Cells were collected by centrifugation, resuspended in PBS with protease inhibitors and lysed. Vimentin was purified as described^33^. Briefly, the lysate was centrifuged and the insoluble fraction resuspended in 20 ml Triton X100, 200 mM NaCl, 10 mM EDTA, 50 mM Tris-Cl, pH 8, homogenized and re-centrifuged three times. The pellet was resuspended in 10 mM EDTA, 50 mM Tris-Cl, pH 8, homogenized and re-centrifuged. After resuspension in 8 M urea, 200 μM EDTA, 5 mM DTT, 200 mM Tris-Cl, pH 8 and incubated overnight at 4 °C, the solubilized vimentin was collected by centrifugation (27,216*g* for 30 min at 4 °C) and stored at −80 °C. For NMR experiments urea was removed from the samples by step-wise dialysis into 4 M urea, 5 mM DTT, 10 mM Tris-Cl, pH 8, followed by 2 M urea, 5 mM DTT, 10 mM Tris-Cl, pH 8 and finally 10 mM Tris-Cl, pH 7.

DNA encoding vimentin rod residues 99–249, cloned into vector pET21a+, were expressed in BL21 (DE3) cells. The vimentin^ROD^ protein was soluble and purified using nickel affinity and size exclusion chromatography with the His tag retained for SPR experiments. D112K and D119K mutant vimentin^ROD^ proteins were purified in a similar fashion. A vimentin^ROD^ double mutant (D112K/D119K) was insoluble and was purified by the refolding protocol described for full-length vimentin.

### Crystallization

Initial conditions for crystallization of the envoplakin PRD were identified using sparse matrix screening. Crystals were grown by sitting-drop vapour diffusion methods with a 3-μl drop volume, containing 1 μl of protein sample (at 6.6 mg ml^−1^) and 2 μl of reservoir solution. The optimal reservoir solution consisted of 20% PEG 3350 and 100 mM bis Tris-Cl, pH 6.5. The iodine derivative of envoplakin PRD was prepared by soaking a single crystal in mother liquor supplemented with an increasing concentration of sodium iodide up to a maximum concentration of 350 mM for 30 min.

### Data collection and processing

Before data collection, the native and iodine derivatized envoplakin PRD crysals were soaked in mother liquor incorporating increasing concentrations of up to 18% ethylene glycol before being flash cooled at 100 K under a nitrogen gas stream (Oxford Cryosystems). Diffraction data were collected using an in-house MicroMax 007HF rotating anode X-ray generator (Rigaku) with a Saturn CCD detector. The native envoplakin PRD crystal diffracted X-rays to 1.6 Å resolution and crystallized in the orthorhombic space group P2_1_2_1_2_1_ with unit cell parameters (*a*=42.4 Å, *b*=68.8 Å, *c*=112 Å, *α*=*β*=*γ*=90°). The iodine-derivatized envoplakin PRD crystal diffracted X-rays to 2.2 Å resolution and was isomorphous to the native crystal belonging to the orthorhombic space group P2_1_2_1_2_1_ with unit cell parameters (*a*=42.3 Å, *b*=68.5 Å, *c*=113.7 Å, *α*=*β*=*γ*=90°), suggesting that iodide soaking had little effect on the internal crystal packing and space group. All diffraction data were integrated, scaled and merged using programs of the XDS suite^34^. The relevant data-processing statistics are listed in Table 1.

### Structure determination

Since initial attempts for determining phases with molecular replacement using desmoplakin PRD-C and PRD-B (PDB codes: ILM5 and ILM7, respectively) failed, the structure of envoplakin PRD was solved by the single-wavelength anomalous diffraction with iodine anomalous signals. In total, 15 iodine sites were located using phenix.hyss and the initial single-wavelength anomalous diffraction phases were calculated by Phenix.Autosol^35^. The anomalous substructure was refined and extended, and phases were estimated using PHASER^36^ followed by density improvement in RESOLVE^37^. Initial models were built using Phenix.autobuild^38^. The final model was produced after several iterative rounds of manual re-building in COOT^39^ and refinement in PHENIX. The final refinement statistics are listed in Table 1. The stereochemical quality of the protein structure was verified using the program PROCHECK^40^. All non-glycine residues are found in the allowed regions of the Ramachandran plot. The final model contains two molecules (a and b) in the asymmetric unit which is comprised of residues D1822-A2014. Structure figures were produced using Pymol (The PyMOL Molecular Graphics System, Version 1.5.0.4 Schrödinger, LLC), with the potential electrostatic surface of the envoplakin PRD being calculated using DelPhi^41^. All structure comparisons were performed using DALI Pairwise^42^ and programs of the CCP4 suite^43^.

### NMR assignment of the envoplakin PRD

Multi-dimensional heteronuclear NMR spectra were recorded at 298 K on a Varian INOVA 600-MHz spectrometer. The experiments were performed using a triple resonance cryogenically cooled probe. The amino-acid sequential assignments of the ^15^N/^13^C-labelled envoplakin PRD were obtained by collecting and analysing a set of triple-resonance experiments, including HNCACB, CBCA(CO)NH, HNCO, C(CO)NH and H(CCO)NH. Spectra were processed with NMRPipe^44^ and analysed using CCPN^45^ and nmrDraw.

### NMR analysis of envoplakin PRD–vimentin binding

All samples analysed by NMR contained 100 μM ^15^N-labelled wild type and mutant envoplakin PRD proteins in 20 mM Tris-Cl, pH 7 and 1 mM DTT. Heteronuclear single quantum coherence spectra were recorded at 298 K on a Varian INOVA 600-MHz spectrometer. The experiments were performed using a triple resonance cryogenically cooled probe. Binding to vimentin was monitored by following the changes in peak intensity on addition of unlabelled vimentin to final concentrations of 10, 50, 100 and 200 μM. NMR chemical shift assignments could be readily transferred between constructs and states due to the relatively minor chemical shift changes.

### SPR analysis of envoplakin PRD–vimentin binding

Envoplakin PRD-vimentin binding was measured following immobilization of His-tagged vimentin^ROD^ on nickel-coated (SA) chips (GE Life Sciences) using a Biacore 3000 machine (GE Life Sciences) and standard protocols. Binding of wild type and mutant envoplakin PRD (0–100 μM) to His-tagged vimentin^ROD^ was measured in the presence of 150 mM NaCl in 10 mM Hepes, 3 mM EDTA, 0.005% Surfactant P20, pH 7.4. Binding of wild-type envoplakin PRD to His-tagged vimentin^ROD^ mutants was measured in a similar fashion. Data was analysed using BIA Evalutation software (GE Life Sciences).

### Modelling the envoplakin PRD–vimentin complex

The interaction between envoplakin PRD and vimentin was modelled with HADDOCK^17^. Envoplakin residues were classified as active in vimentin binding based on the results of perturbations of ^15^N-resolved NMR signals, exposure in the conserved basic groove, and mutagenesis experiments while ‘passively involved' residues were selected automatically. Vimentin residues contacted by the PRD were predicted from conservation of sequence motifs and negative charge as well as surface exposure.

To generate representative structural models of the envoplakin PRD–vimentin complex, molecular docking experiments were repeated with all available vimentin structures including PDB entries 1GK4 (ref. 46), 1GK6 (ref. 46), 3G1E (ref. 18), 3KLT (ref. 47), 3SWK (ref. 48), 3TRT (ref. 49) and 3UF1 (ref. 14). Residues selected for use as restraints can be found in Supplementary Table 1, along with the results of the HADDOCK runs. Notably, the docking procedure was performed with both monomeric and dimeric forms of vimentin. The representative structures with the lowest energies were selected for detailed analysis and displayed.

### Transfection, western blotting and pull-down assays

DNA encoding human envoplakin (residues 1542–2014) with a C-terminal Flag tag (DYKDDDDK), and DNA encoding human periplakin (residues 1588–1756) with a C-terminal HA tag (YPYDVPDYA), was cloned into expression vector pcDNA3.1(−) (Life Technologies). Mutant envoplakin constructs were produced using the QuikChange Lightening kit. Constructs were transfected into cultured HeLa cells (supplied by Cancer Research UK, London Research Institute) using GeneJammer transfection reagent (Agilent Technologies). For western blotting transfected cells were lysed in SDS sample buffer, resolved by SDS–polyacrylamide gel electrophoresis and transferred to Hybond-LFP polyvinylidene difluoride membrane. Blots were probed with anti-Flag (1:3,000; Sigma F7425), anti-HA (1:500; Santa Cruz sc-805) and anti-actin (1:20,000; Sigma A5441) antibodies, followed by HRP-conjugated secondary antibodies (1:1,000; Dako P0447/P0448).

Intracellular interactions between plakin proteins were detected in HeLa cells by pull-down assays. At 48 h following transfection cells were lysed in PBS containing 1% Triton X-100, 2 mM phenylmethylsulphonyl fluoride, 10 mg ml^−1^ aprotinin and 10 mg ml^−1^ leupeptin. The insoluble material was pelleted by centrifugation at 13,000 r.p.m. for 10 min at 4 °C and Flag-agarose beads (Sigma) added to the supernatant. The tubes were mixed by inversion on a rotating wheel for 45 min at 4 °C, the beads were then washed repeatedly and bound proteins eluted with SDS sample buffer. Proteins were separated by electrophoresis, blotted onto hybond membrane and probed with anti-Flag and anti-HA antibodies. Uncropped scans of blots showing expression of envoplakin and periplakin in lysates, and the results of pull-down assays are shown in Supplementary Fig. 8.

### Immunofluorescence staining

HeLa cells were grown on glass coverslips in complete media for 24–36 h before transfection with appropriate DNA plasmids using GeneJammer reagent. At 48 h following transfection cells were fixed for 10 min in 2% paraformaldehyde and permeabilized for 2 min with 0.1% Triton X-100. Cells were co-stained with either anti-Flag (1:1,000) or anti-HA (1:1,000) and either anti-vimentin (1:75; Cell Signaling #3932) or anti-keratin 8 (1:1,000; Sigma C5301) antibodies, followed by AlexaFluor-conjugated secondary antibodies (1:1,000; Molecular Probes A-11017/A-11019/A-21069/A-11070). Coverslips were mounted onto microscope slides using SlowFade Gold antifade reagent (Life Technologies). Images were taken using Zeiss LSM510 META confocal system with × 63 oil immersion objective (NA 1.4).

### AUC

Sedimentation equilibrium experiments were carried out using a Beckman XL-1 analytical ultracentrifuge (Beckman Coulter, Palo Alto) with an eight-cell 50Ti rotor. Samples were prepared in 100 mM NaCl, 20 mM sodium phosphate, pH 7.4 with three protein concentrations between 0.05 and 4 mg ml^−1^ being loaded into double sector cells and centrifuged at 35,000, 37,000 and 39,000 r.p.m. for 20 h at 20 °C. Sedimentation coefficients and molecular masses were determined using the continuous *c(s)* analysis method and SEDFIT software^50^. Sedimentation velocity experiments were carried out in the Beckman XL-1 centrifuge with a An50Ti rotor using a double sector cell with a 12 mm Epon charcoal-filled centre piece. Initial checks were performed at 3,000 r.p.m. The sample (0.5 mg ml^−1^ in 100 mM NaCl, 20 mM sodium phosphate, pH 7.4) was then centrifuged at 40,000 r.p.m. for 12 h at 20 °C.

### SAXS

SAXS data were collected at the X33 beamline, EMBL Hamburg outstation as described^51^. Scattering patterns were collected at room temperature using purified protein at concentrations between 1 and 5.5 mg ml^−1^ in 100 mM NaCl, 20 mM sodium phosphate, pH 7.2. Given that the 5.5 mg ml^−1^ data set gave the highest signal to noise, the other data sets were discarded, and the 5.5 mg ml^−1^ data set was subsequently used for the final analysis. Background scattering from buffer alone was automatically subtracted from the protein scattering profiles using the program PRIMUS^52^. An average of the final *ab initio* model of the envoplakin PRD was created using the following steps. Using the data range of 0–0.3 Å^−1^ the data was analysed by GNOM^53^ and the *R*_g_ and *I*_0_ values calculated. The GNOM output file was fed into the program DAMMIF^54^ that generated 10 models. These models were aligned and compared using the programs DAMSEL and DAMSUP. The outputs of these programs were fed into DAMAVER^55^, which created an averaged model of the original 10 models. Finally, extraneous regions were filtered by DAMFILT, which gave the final model. The data was compared against the crystal structure using CRYSOL^56^.

### Far ultra-violet circular dichroism spectroscopy

CD spectra were measured on a JASCO J-1500 spectrometer using a 1-cm path length cuvette and a protein concentration of 0.7 μM. The scanned wavelength range was 190–260 nm. The secondary structure content was estimated from the CD spectra using the CDSSTR algorithm and reference data set 7 on the DichroWeb server^15^,^16^.

## Additional information

**Accession codes:** The atomic co-ordinates and experimental data for the envoplakin PRD have been deposited in the Protein Data Bank (4QMD).

**How to cite this article:** Fogl, C. *et al*. Mechanism of intermediate filament recognition by plakin repeat domains revealed by envoplakin targeting of vimentin. *Nat. Commun.* 7:10827 doi: 10.1038/ncomms10827 (2016).

## Supplementary Material

Supplementary Information Supplementary Figures 1-9, Supplementary Table 1 and Supplementary References

## Figures and Tables

**Figure 1:**
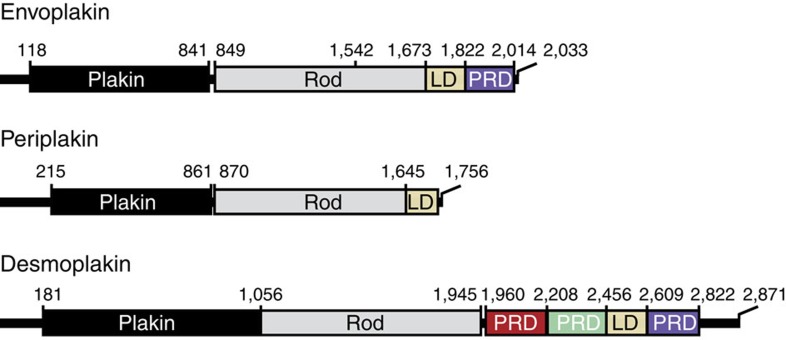
Domain architecture of plakin family members. Envoplakin contains an N-terminal plakin domain, a central rod domain that forms a helical coiled coil, and a C-terminal tail region consisting of a linker domain (LD) and a single plakin repeat domain (PRD). Periplakin lacks a PRD module whereas desmoplakin possesses three. The domain boundaries are indicated.

**Table 1:** Data processing and refinement statistics for the envoplakin PRD.

**Parameters**	**Envoplakin–PRD**	**Envoplakin–PRDi**
*Data collection*		
Space group	P2_1_2_1_2_1_	P2_1_2_1_2_1_
Cell dimensions		
*a*, *b*, *c* (Å)	42.4, 68.8, 112.2	42.3, 68.5, 113.7
*α*, *β*, *γ* (°)	90.0, 90.0, 90.0	90.0, 90.0, 90.0
Resolution (Å)	20–1.6 (1.7–1.6)	20–2.2 (2.3–2.2)
*R*_sym_ or *R*_merge_	5.8 (38.5)	10.6 (37.9)
*I*/*σI*	27.9 (2.1)	32 (10.9)
Completeness (%)	92.9 (67.2)	99.8 (99.9)
Redundancy	6.9 (2.1)	23.9 (23.5)
		
*Refinement*		
Resolution (Å)	19.9–1.6	
No. reflections	41,953	
*R*_work_/*R*_free_	16.8/20.5	
No. atoms		
Protein	2,979	
Ligand/ion	—	
Water	441	
B-factors (Å^2^)		
Protein	11.1	
Ligand/ion	—	
Water	26.3	
R.m.s. deviations		
Bond lengths (Å)	0.006	
Bond angles (°)	1.038	

PRD, plakin repeat domain.

Values in parentheses apply to data in the highest resolution shell. For both data sets Friedel's pairs are treated as independent reflections. Native and iodine derivative X-ray data were collected from single crystals.
