# Supplementary material for: Mechanism of intermediate filament recognition by plakin repeat domains revealed by envoplakin targeting of vimentin
Source: Nat Commun. 2016 Mar 3;7:10827. doi: 10.1038/ncomms10827 (PMC4782060; doi:10.1038/ncomms10827)
Supplement: Supplementary Information — Supplementary Figures 1-9, Supplementary Table 1 and Supplementary References [file ncomms10827-s1.pdf]

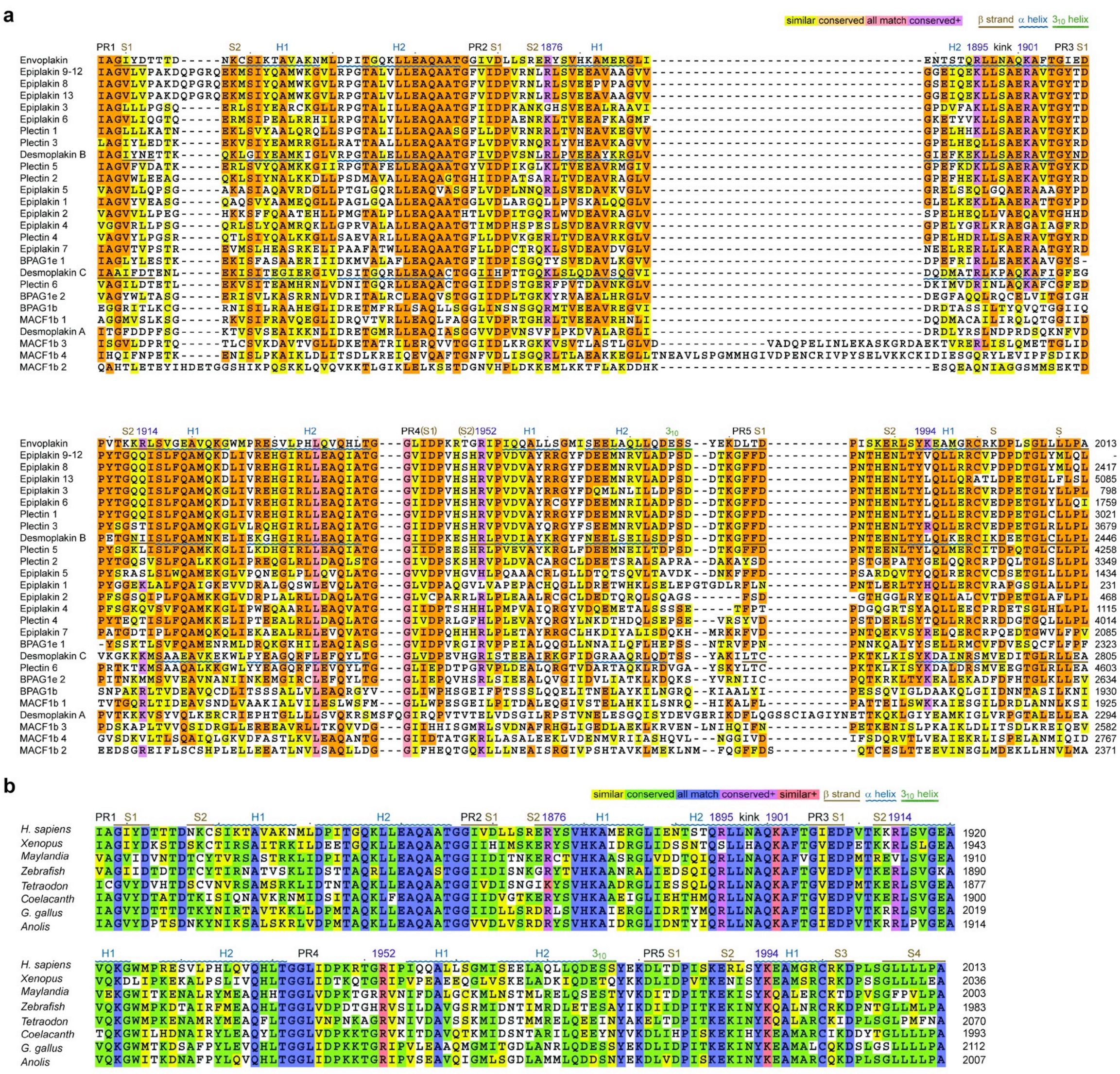

Supplementary Fig. 1 Multiple sequence alignment of PRD domains. (a) Alignment of PRD sequences from the human plakin proteins. Identical, conserved and similar residues are coloured red, orange and yellow respectively, while the critical conserved and similar basic residues are in purple. Known secondary structure elements are indicated under the residues. All alignments were produced using the ClustalW 1.6 similarity matrix and corrected to align structural elements (1). (b) Alignment of envoplakin PRD sequences from different vertebrate species with identical, conserved and similar residues coloured blue, green and yellow respectively, and the critical conserved and similar basic residues in purple and red. The secondary structural elements are indicated above the human envoplakin sequence.

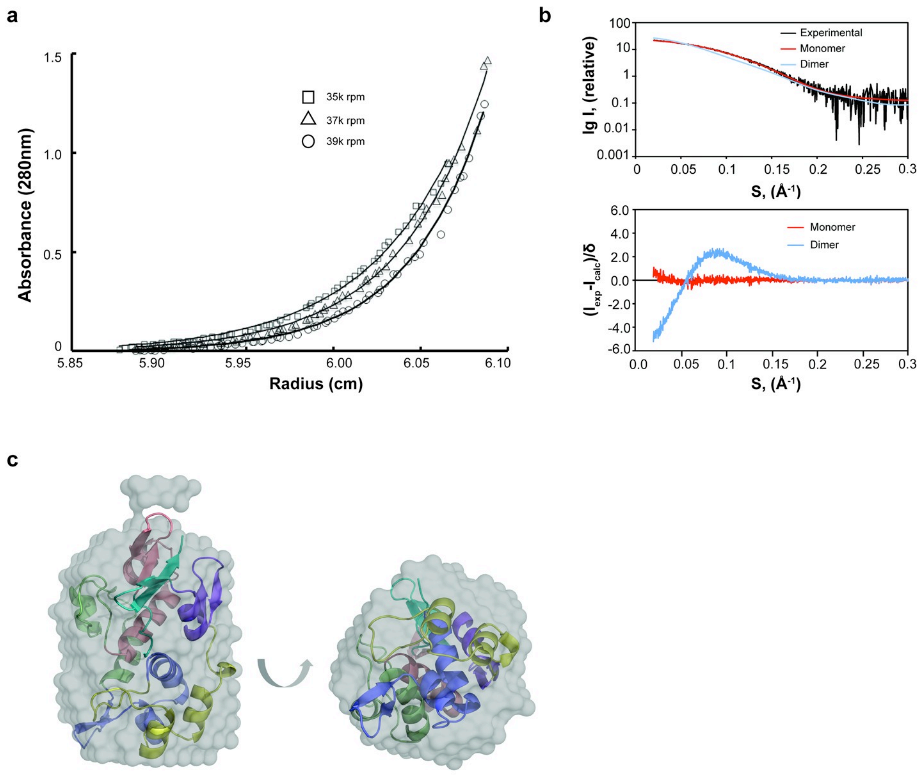

Supplementary Fig. 2 The envoplakin PRD is monomeric by AUC analysis and small angle X-ray scattering. (a) Sedimentation equilibrium AUC of the envoplakin PRD. The data is shown for the highest concentration of envoplakin PRD (65 µM) and confirms that the protein is monomeric. The calculated molecular weight is 20.1 kD which compares well with the theoretical molecular weight of 21.9 kDa. (b) Experimental SAXS scattering data for the envoplakin PRD with CRYSOL fit calculated from the putative monomeric and dimeric structures. The residual plot indicates the level of corroboration between the experimental scattering data and the CRYSOL fit, and show that the envoplakin PRD is a monomer and not a dimer as shown in the crystal lattice. χ value for the monomeric fit was 1.02. For each concentration used at the synchrotron (1.0, 3.7 and 5.0 mg/ml) the Rg and I_0 values were calculated using the program GNOM. Values were similar at all concentrations indicating that the envoplakin PRD was not aggregated. (c) The crystal structure was automatically inserted into the final averaged SAXS model using the program SUPCOMB (2). Individual plakin repeats are coloured as in Fig. 2.

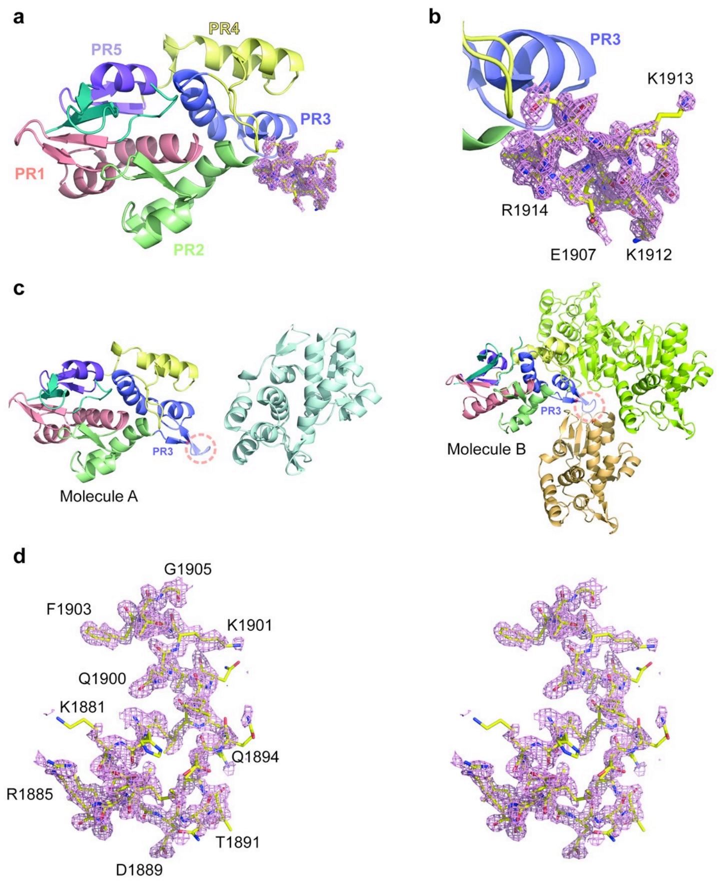

Supplementary Fig. 3 Definition of functionally relevant regions of the envoplakin PRD. (a) Envoplakin PRD (coloured as in Fig. 2a) with the 1.6Å resolution composite omit *Fo-Fc* electron density map for the envoplakin PR3 β-hairpin is contoured at 1.5σ (pink). (b) Close-up view of the envoplakin PR3 β-hairpin region shown in (a). (c) Crystal lattice environment for the envoplakin PR3 β-hairpin region. The envoplakin PR3 β-hairpin of molecule A and B (red circles) participate in crystal contacts with symmetry related molecules (coloured in cyan, orange and limon) (d) Stereoview of the composite omit *Fo-Fc* electron density map (pink) contoured at 1.5σ for the PR2 helical kink.

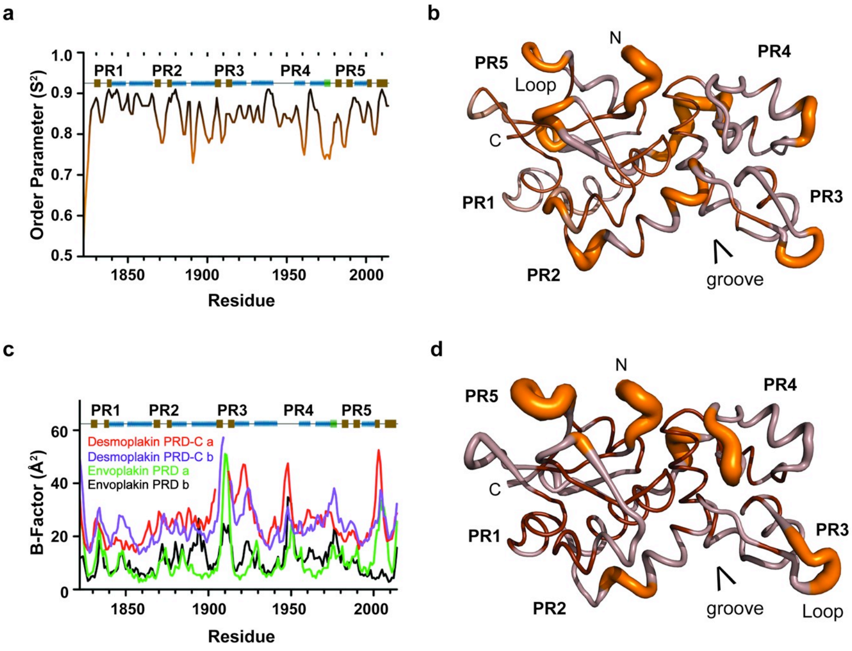

Supplementary Fig. 4 Dynamics of the envoplakin PRD. (a) The dynamics of the PRD backbone are indicated by the order parameter (S^2) of each residue as calculated using the TALOS$^+$ server (12). (b) Backbone ribbon of the envoplakin PRD, where the width of the ribbon is inversely related to the order. Orange is used for regions with greater disorder, beige for moderate disorder and brown for little disorder. (c) The graph shows the backbone B-factors of the desmoplakin and envoplakin PRD structures, except for the desmoplakin PR3 motif which is not defined. (d) Backbone ribbon of the r.m.s. deviations between Cα atoms for the two envoplakin PRD molecules found in the asymmetric unit. Orange thick sections indicate regions of the greatest deviation, beige for medium difference and brown for areas which superimpose closely.

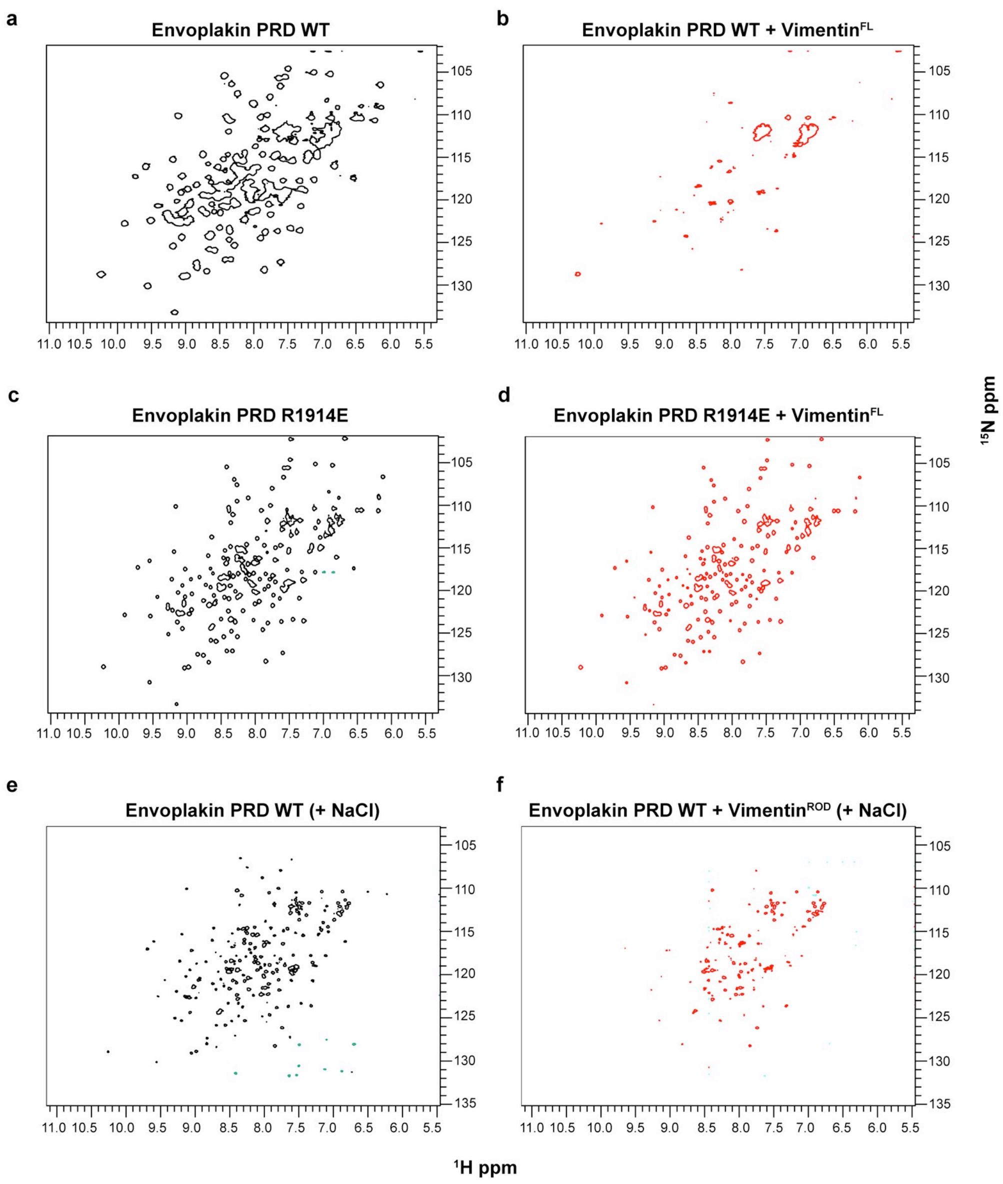

Supplementary Fig. 5 Binding of the envoplakin PRD to vimentin^{FL} and vimentin^{ROD} proteins. (a) ^{15}N HSQC spectrum of 100 µM envoplakin PRD (black) reveals 184 dispersed resonances as expected for its mixed α/β structure of 193 residues. (b) HSQC spectrum of 100 µM envoplakin PRD plus 50 µM vimentin^{FL} (red) showing a significant loss of peaks. (c) HSQC spectrum of 100 µM envoplakin PRD R1914E (black) showing a similar number of peaks and dispersion pattern as wild type envoplakin PRD. (d) HSQC spectrum of 100 µM envoplakin PRD R1914E plus 50 µM vimentin^{FL} (red) showing no peak loss in contrast to the wild type envoplakin PRD where most peaks are lost. (e) HSQC spectrum of 100 µM envoplakin PRD (black) in the presence of 150 mM NaCl. (f) ^{15}N HSQC spectrum of 100 µM envoplakin PRD plus 50 µM vimentin^{ROD} (red) in the presence of 150mM NaCl, showing a significant loss of peaks. Experiments with vimentin^{FL} were carried out in the absence of NaCl.

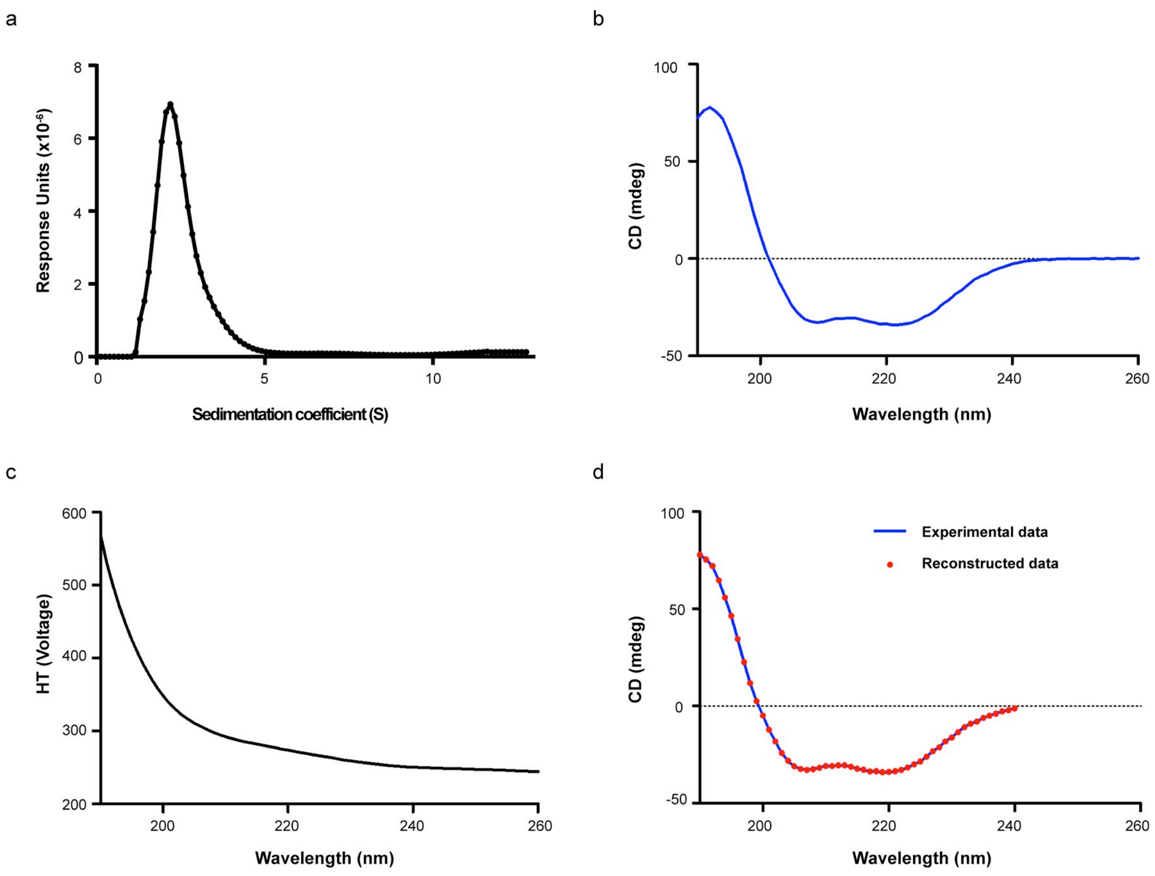

Supplementary Fig. 6 The vimentin^{ROD} is monomeric with a high α-helical content. (a) Sedimentation velocity AUC of vimentin 99-249. The experiment was carried out with 28 μM protein and confirms that vimentin 99-249 is monomeric. (b) Circular dichroism spectrum of 0.7μM Vimentin 99-249 in 10mM sodium phosphate buffer pH 7.4. Spectrum recorded on a JASCO J-1500 spectrometer using 1cm path length, 1nm data interval, 1nm data pitch, 1nm bandwidth and a scanning speed of 50nm/min. (c) The change in high tension voltage as a function of wavelength. (d) Fit of the CD spectrum using the CDSSTR algorithm and reference dataset 7 on the DichroWeb web server.

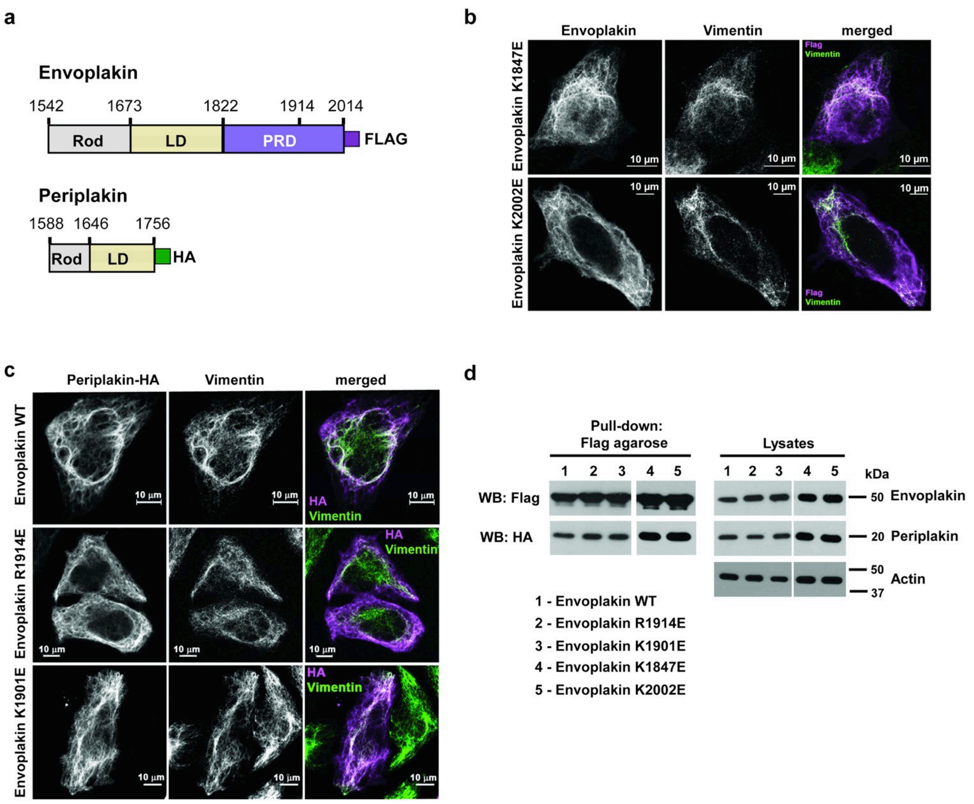

Supplementary Fig. 7 Transfection of envoplakin and periplakin C-terminal constructs into HeLa cells. (a) Diagram showing the envoplakin and periplakin constructs used in transfection experiments. (b) Envoplakin mutants K1847E and K2002E show identical patterns of staining to wild-type envoplakin. (c) Periplakin shows extensive co-localisation with vimentin intermediate filaments when transfected with either wild-type, R1914E and K1901E mutant envoplakins. (d) Expression of wild-type and mutant envoplakins is similar by western blotting and both wild-type and mutant envoplakins interact with periplakin in pull-down assays. Transfected cells were lysed and Flag tagged envoplakin pulled down using Flag-agarose beads. The beads were washed and bound proteins eluted from the beads, resolved by SDS-PAGE and blotted onto nitrocellulose. Blots were probed with anti-Flag and anti-HA antibodies (against periplakin).

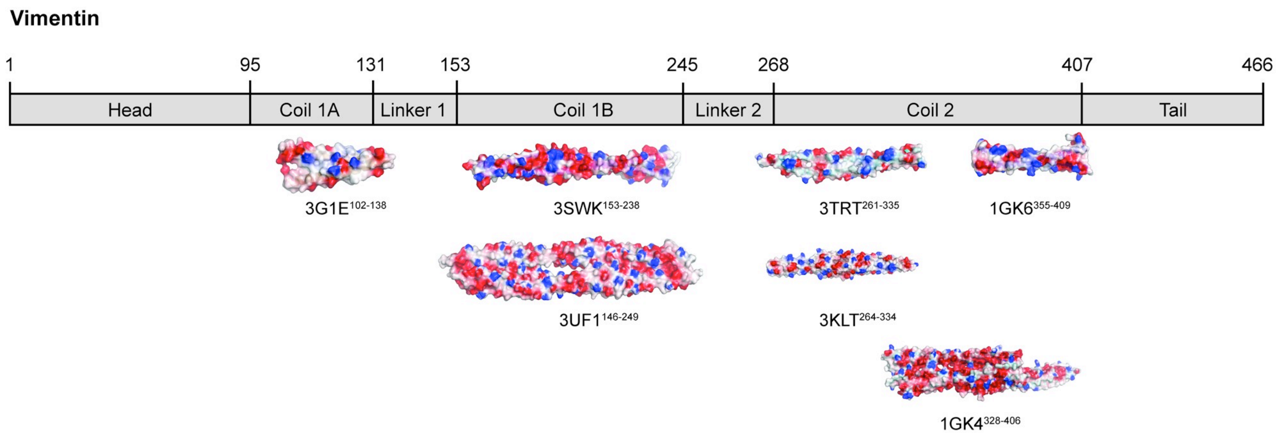

Supplementary Fig. 8 Domain architecture of vimentin. Vimentin encompasses a highly α-helical central domain, with flanking non-helical 'head' and 'tail' domains. Electrostatic potential representations of the vimentin fragments using in HADDOCK docking with the envoplakin PRD are shown. The electrostatic potential of the vimentin structures, which are shown with their PDB accession numbers, was calculated with DelPhi with the potential scale ranging from -7 (red) to +7 (blue) in units of kT/e.

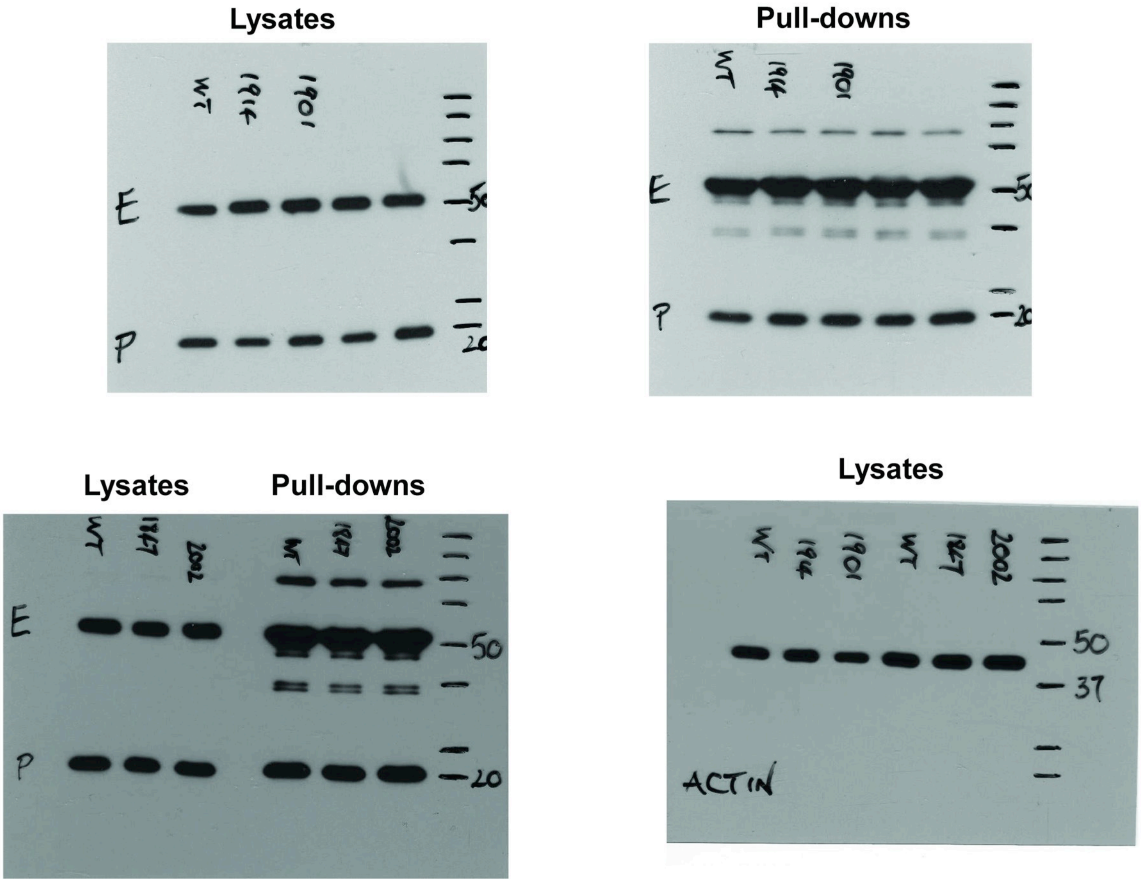

Supplementary Fig. 9 Uncropped scans of blots showing expression of envoplakin and periplakin in lysates, and the results of pull-down assays. These data are presented in Supplementary Fig. 6d.

Supplementary Table 1 Summary of docking results for envoplakin PRD and different vimentin structures using HADDOCK

Vimentin PDB Entry	Vimentin Residues	HADDOCK score	Cluster size	Restraints violation energy	Overall RMSD (Å)	Buried surface area (A^2)	Vimentin restraints
3G1E	N102-L138	-93.1 ± 5.9	27	101.7 ± 33.32	1.2 ± 41.5	1536.5 ± 41.5	E103, D119, E125
3SWK	E153-H238	-92.6 ± 8.0	45	72.5 ± 45.56	2.4 ± 0.8	1229.5 ± 153.8	E192, D200, D211, D219, E225, E230
1GK6	M355-S409	-92.4 ± 11.7	21	83.7 ± 3.01	1.0 ± 0.6	1318.9 ± 61.6	E396, E407, E408
1GK4	C328-G406	-75.0 ± 2.0	131	12.0 ± 20.78	1.2 ± 0.7	1014.2 ± 126.1	E396, E407, E408
3UF1	L146-I249	-70.7 ± 14.4	8	116.5 ± 45.53	1.3 ± 0.7	1362.0 ± 151.6	E192, D200, D211, D219, E225, E230, E241
3TRT	S261-G335	-66.9 ± 7.5	19	21.9 ± 22.08	18.6 ± 0.8	1442.3 ± 71.7	D264, E277, E288
3KLT	D264-K334	-60.9 ± 10.1	5	93.9 ± 54.21	21.0 ± 0.2	1231.2 ± 158.0	D264, E277, E288

Haddock statistics are listed for the top cluster of each envoplakin PRD-vimentin model. Energies are expressed in kcal mol^{-1}. RMSD is the root mean square deviation from the overall lowest energy structure. Conserved vimentin residues selected for ambiguous intermolecular restraints are also shown.

Supplementary References

1. Larkin M. A., et al. Clustal W and Clustal X version 2.0 *Bioinformatics* **23**, 2947-2948 (2007)

2. Kozin M. B. & Svegun D. I. Automated matching of high- and low-resolution structural models. *J. Appl. Cryst.* **34**, 33-41 (2001)
